# Mixed methods grant applications in the health sciences: An analysis of reviewer comments

**DOI:** 10.1371/journal.pone.0225308

**Published:** 2019-11-15

**Authors:** Timothy C. Guetterman, Rae V. Sakakibara, Vicki L. Plano Clark, Mark Luborsky, Sarah M. Murray, Felipe González Castro, John W. Creswell, Charles Deutsch, Joseph J. Gallo

**Affiliations:** 1 Graduate School, Creighton University, Omaha, Nebraska, United States of America; 2 Department of Family Medicine, University of Michigan, Ann Arbor, Michigan, United States of America; 3 School of Education, University of Cincinnati, Cincinnati, Ohio, United States of America; 4 Institute of Gerontology, Wayne State University, Detroit, Michigan, United States of America; 5 Department of Mental Health, Johns Hopkins Bloomberg School of Public Health, Baltimore, Maryland, United States of America; 6 College of Nursing and Health Innovation, Arizona State University, Phoenix, Arizona, United States of America; 7 Harvard Catalyst, Harvard University, Boston, Massachusetts, United States of America; Charles P. Darby Children's Research Institute, 173 Ashley Avenue, Charleston, SC 29425, USA, UNITED STATES

## Abstract

Our aim was to understand how reviewers appraise mixed methods research by analyzing reviewer comments for grant applications submitted primarily to the National Institutes of Health. We requested scholars and consultants in the Mixed Methods Research Training Program (MMRTP) for the Health Sciences to send us summary statements from their mixed methods grant applications and obtained 40 summary statements of funded (40%) and unfunded (60%) mixed methods grant applications. We conducted a document analysis using a coding rubric based on the NIH Best Practices for Mixed Methods Research in the Health Sciences and allowed inductive codes to emerge. Reviewers favorably appraised mixed methods applications demonstrating coherence among aims and research design elements, detailed methods, plans for mixed methods integration, and the use of theoretical models. Reviewers identified weaknesses in mixed methods applications that lacked methodological details or rationales, had a high participant burden, and failed to delineate investigator roles. Successful mixed methods applications convey assumptions behind the methods chosen to accomplish specific aims and clearly detail the procedures to be taken. Investigators planning to use mixed methods should remember that reviewers are looking for both points of view.

## Introduction

Mixed methods research is defined as the collection, analysis, and integration of both quantitative data (e.g., RCT outcome) and qualitative data (e.g., observations, semi-structured interviews) to provide a more comprehensive understanding of a research problem than might be obtained through quantitative or qualitative research alone.[1] Relevant strategies for the use of mixed methods in health services research include adding qualitative interviews to follow up on the outcomes of intervention trials, gathering both quantitative and qualitative data to assess patient reactions to a program implemented in a community health setting, or using qualitative data to describe or explain the mechanism of a study correlating behavioral and social factors to specific health outcomes.[2, 3] In multilevel behavioral interventions, investigators need to qualitatively understand context to develop a more complete picture of how implementation is occurring.[4] While quantitative approaches can characterize and measure patient outcomes, use of mixed methods can enhance quantitative analyses to identify unmeasured factors that might be associated with poor responses to an intervention,[5] factors that can account for people who do not “fit the model” (i.e. outliers),[6] hypotheses on how interventions can be designed for greater responsiveness to the needs and circumstances of diverse cultural groups (e.g., racial/ethnic minority individuals), or adaptations needed in interventions for the unique needs of special cases (e.g., “personalized interventions”).[7]

An increase in the use of mixed methods within proposals submitted to the National Institutes of Health (NIH)[8, 9] and in health services research journals[10] reflects the growing awareness of the importance of this approach in addressing population and behavioral health. In 2011, in response to increasing numbers of applications to NIH employing mixed methods, the Office of Behavioral and Social Science Research convened a panel to provide guidance to investigators writing mixed methods proposals,[11] which the NIH later updated in 2018.[12] The resulting *Best Practices for Mixed Methods Research in the Health Sciences* includes a set of questions to improve the quality of mixed methods applications keyed to the criteria used by NIH study sections to evaluate proposals (namely, Significance, Investigators, Innovation, Approach, and Environment). A limitation of this and other guidance available for writing mixed methods proposals[13–16] is that none are informed by empirical analysis of critical reviews of submitted mixed methods proposals.

To address this gap, the aim of this study was to understand how reviewers appraise mixed methods research by analyzing reviewer comments for grant applications submitted primarily to the NIH. For many investigators, particularly in health services research involving intervention development and implementation science, successful applications to NIH at the R- and K-series are essential to support research and career development. We leveraged the network of scholars and consultants in the NIH-funded Mixed Methods Research Training Program (MMRTP) for the Health Sciences to obtain summary statements of both funded and unfunded mixed methods proposals. We then analyzed the content of these documents to examine the strengths and weaknesses of mixed methods in NIH proposals as identified by study section reviewers.

## Methods

### Mixed Methods Research Training Program

The context for our study was the MMRTP, an NIH-funded training grant designed to provide intensive training in mixed methods research to scholars through the development of an NIH grant proposal. Competitively selected scholars (primarily early-career faculty) participate in 1) a three-day in-person retreat with lectures and interactive discussions about their projects, 2) webinars on mixed methods topics, and 3) ongoing support from a mentor selected from a network of consultants created for the MMRTP who each have content and mixed methods expertise.[17–19]

### Recruitment procedure

We contacted 74 MMRTP scholars and consultants via email to request summary statements of funded and unfunded mixed methods proposals. Participation was voluntary. Twenty-two scholars sent copies of relevant summary statements to form the database for the document analysis. The Institutional Review Board of the Johns Hopkins Bloomberg School of Public Health deemed the study exempt as educational research.

### Analysis strategy

Our focus in analyzing the summary statements was on reviewer comments specific to mixed methods aspects. The NIH Best Practices for Mixed Methods Research in the Health Sciences[11, 12] provided a rubric to code summary statements. Domains of the *Best Practices* guidance for review follow NIH criteria; namely, ‘Significance,’ ‘Investigators,’ ‘Innovation,’ ‘Approach,’ and ‘Environment,’ but with specific mixed methods features for each (and modified criteria for K mentored scientist awards). Each criterion was used as an *a priori* code; however, in addition to this deductive coding approach, we also used inductive coding,[20] identifying new themes based on reviewers’ comments. Using MAXQDA software version 18 (Berlin, Germany), we applied codes to relevant segments from each summary statement and generated queries to examine patterns across grant applications. As a guide for readers, we paid special attention to the number and content of codes representing reviewer comments about funded and unfunded applications, keeping in mind that reviewers do not make funding decisions, and many factors are considered in funding decisions. We have selected illustrative quotes verbatim, but redacted potentially identifiable information to maintain anonymity.

## Results

### Study sample

A total of 41 summary statements of mixed methods grant applications were collected from 22 MMRTP scholars and consultants for a response rate of 29.7% (Table 1). One review document was omitted from analysis because it was not written in English. No summary statements were shared for applications that were ‘not discussed,’ i.e. whether funded or non-funded, all summary statements were for grant applications that scored in the top half of submitted proposals for a given study section meeting. The time period covered by the grant applications in our sample ranged from 1997 to 2017. Half of the applications were submitted in or after 2015. Most applications were first submissions (62.5%) rather than resubmissions (37.5%).

**Table 1:** Characteristics of the sample of reviews from mixed methods proposals.

Total Grant Reviews (*N* = 40)		Funding Institutes	
Document Type		**National Institute of Health**	
Summary Statements	36	National Institute of Drug Abuse	7
Letters to Reviewers	3	National Institute of Mental Health	7
Internal Reviews	1	National Institute of Child Health and Human Development	5
Funding		National Cancer Institute	4
Unfunded	24	National Institute of Diabetes and Digestive and Kidney Diseases	3
Funded	16	National Institute on Aging	3
Grant Type		National Heart, Lung, and Blood Institute	2
Research Grant (R)	29	National Institute of Neurological Disorders and Stroke	2
Career Development (K) Grant	7	National Institute of Nursing Research	2
Non-NIH Research Grant	4	**Other Agencies**	
Application Type		Patient-Centered Outcomes Research Institute	2
First Submission	25	Agency for Healthcare Research Quality	1
Re-submission (A1)	13	Pharmaceutical Company	1
Second Re-submission (A2)	2	University-associated Clinical and Translational Science Institute	1

In our sample, 40% of applications were funded. Criteria mean scores and standard deviations for each review criterion (Significance, Investigators, Innovation, Approach, Environment) from NIH summary statements (n = 33) are provided in Table 2. Mean scores across criteria were rather favorable in both funded R- and K-series applications (1.75 and 1.47, respectively) and unfunded applications (2.75 and 1.77, respectively). As expected, scores were less favorable for unfunded applications. In both funded and unfunded research grant applications, the Approach section had the highest (least favorable) mean scores (2.62 and 4.27, respectively) compared to other review criteria. Likewise, for both funded and unfunded career development award applications, the corresponding Research Plan section had the highest (least favorable) mean scores (2.42 and 3.17, respectively).

**Table 2:** Criterion mean scores (SD) in summary statements of R- and K-series mixed methods proposals to NIH.

**R Series Review Criteria**	Funded	Unfunded
Significance	1.90 (1.00)	2.94 (1.34)
Investigator(s)	1.29 (.46)	2.10 (1.24)
Innovation	1.71 (.72)	2.77 (1.60)
Approach	2.62 (1.12)	4.27 (1.74)
Environment	1.24 (.44)	1.69 (.97)
**K Series Review Criteria**		
Candidate	1.25 (.45)	1.17 (.41)
Career Development Plan/ Career Goals/Plan to Provide Mentoring	1.50 (.91)	2.17 (1.17)
Research Plan	2.42 (1.00)	3.17 (.41)
Mentor(s), Co-Mentors(s), Consultant(s), Collaborators	1.08 (.29)	1.33 (.52)
Environment and Institutional Commitment to the Candidate	1.08 (.29)	1.00 (0)

### The approach or research plan sections

The bulk of reviewer comments relevant to mixed methods research were concentrated in the Approach or Research Plan sections. We combined our analysis of the Approach section for R-series applications and the Research Plan for K-series applications because both address the methods for the research design. Table 3 compares strengths and weaknesses for the Approach (R) and Research Plan (K) sections identified by reviewers for both funded and unfunded grants. Reviewer comments focused on the need for (1) a rationale and coherence among the aims, the research design, and the methods; (2) provision of a detailed description of methods, including the specific procedures for integration of qualitative and quantitative components; and (3) the use of theoretical models or conceptual frameworks.

**Table 3:** Reviewer comments of Approach (R) or Research Plan (K) criterion for mixed methods proposals by reviewer critique focus areas.

Reviewer Critique Focus Areas	Strengths	Weaknesses
*Rationale and coherence among aims*, *design*, *and methods*	[a strength is] “the use of a mixed-methods design to complement quantitative data with richer information about the context of social media use” (Unfunded) “The sample size is clearly driven not by the quantitative analyses, but by the demands of the sampling stratification scheme which in turn is driven by the research questions” (Funded) “The methods and analytic plan were tailored to each of the specific aims” (Unfunded)	“Given the strength of the qualitative component of the study with its elegant design, one questions why the investigators want to include a second and less detailed quantitative dimension” (Funded) “… what is the plan for combined use of the data to answer the key research questions?” (Funded) “More importantly, the hypotheses for each aim are vague. How the qualitative results will inform and impact on quantitative results is not discussed clearly” (Unfunded)
*Provision of detailed description of methods and integration*	“Another strength of the application includes the staff training to assure rigor in interviewing, standardizing project procedures, and sound data management” (Funded) “Qualitative measures are developed in draft form, included in the appendices, and track to key questions related to sustainment. Qualitative research processes are well developed” (Unfunded) “The application is distinguished by the careful specification and integration of methods and analyses with the aims of the project” (Funded)	“Although they mentioned that interviews will be conducted, it is not very clear how such complex data will be analyzed and reported, and how valid and useful the results can be to help guide future practice” (Funded) “Poor research plan and use of qualitative and mixed methods methodology is not clear. No details were provided as it relates to the specific techniques” (Funded) “How will research be triangulated? Comparing and relating is mentioned but need greater detail of how findings will be integrated” (Unfunded)
*Use of theoretical models or conceptual frameworks*	“This revised application now incorporates a theory-driven conceptual model. This constitutes a significant strength” (Funded) “The mixed method approach is well designed and supported by preliminary data and an appropriate conceptual framework” (Unfunded)	“The model, while a good choice, is not laid out in detail” (Funded) “Various sections seem disjointed and lack theoretical connectivity” (Unfunded)

Reviewers commented on coherence between the aims of the proposed study and the mixed methods design used and its description. Moreover, as a way to enhance coherence, reviewers commented on well-developed conceptual models along with detailed descriptions of methods as strengths. Reviewers noted strengths when the integration of qualitative and quantitative methods was clear. Whereas most of these comments were general in nature (“seamless integration of qualitative and qualitative findings represents another virtue of this meritorious application”), a few reviewers praised investigators when integration occurred throughout the entire course of the study. Another noteworthy comment regarding integration came from a reviewer who commended the investigator for recognizing the inductive nature of qualitative analysis and including possible findings informed by the investigator’s prior work. Reviewers perceived strengths when theoretical or conceptual models informed the Approach or Research Plan.

A major concern was the lack of detail about the methods proposed, such as missing descriptions of integration strategies. Specific integration issues raised by reviewers were use of jargon without a clear explanation of what integration means in terms of techniques and procedures, and poor explanation of the linkage between qualitative and quantitative components, namely how the qualitative findings would inform intervention or instrument development. An additional concern was the inadequate description of procedures used in the qualitative phase of the research. Reviewers identified weaknesses in the lack of detail about interviews, observations, or focus group procedures and interviewer or facilitator training plans that standardize the data collection process. In the qualitative analysis plan, reviewers noted weaknesses when data coding procedures, use of data analysis software (if any), and how to ensure reliability with more than one coder were not described. A related concern was the presence of a minimal rationale for the qualitative methods selected. Finally, reviewers raised concerns about feasibility, which were mostly related to the ambitious plan of several projects. A feasibility concern was that investigators underestimated the time it would take to complete the qualitative component, which also raised flags about whether enough time and personnel were allocated to interviewing participants.

### The significance, investigators, innovation, and environment sections

#### R-series grants

Our analysis of reviewer comments using the other R-series application review criteria—Significance, Investigators, Innovation, and Environment—revealed additional information about how the use of mixed methods was appraised (Table 4). Interestingly, no notable comments were identified related to the Environment criteria for R grant applications. Regarding Significance, the use of mixed methods to develop a new understanding of phenomena was viewed as a strength, especially when mixed methods had not been previously applied. Weaknesses noted were related to the relevance of the research topics rather than the use of mixed methods. We found no noteworthy differences regarding significance between funded and unfunded applications.

**Table 4:** Reviewer comments for the Significance, Investigators, Innovation, and Environment criteria for R and K-series mixed methods proposals.

Strengths		Weaknesses	
**R-Series Applications**	**K-Series Applications**	**R-Series Applications**	**K-Series Applications**
**Significance**			
“One of the strongest design features of this study is the extent to which the investigators will address their research questions with multiple kinds of information and with multiple strategies of analysis. These multiple directions will add layers of richness to the results that will make them particularly useful” (Funded)	“The use of mixed-methods, including conjoint analysis, to develop understandings of patient factors affecting acceptance of depression treatment, and for tailoring interventions to address patient concerns, is both innovative and significant…” (Unfunded)	None noted	None noted
**Investigators**			
“The role of each co-investigator is appropriate for their expertise, clearly articulated in the application, and distinct (not duplicative among the team)" (Funded)	“Strong prior work in qualitative research methods complements her more quantitatively-oriented education plan, but additional qualitative training is also included” (Unfunded)	“the various contributions of the six Co-Is is not very clearly presented or justified and there appears to be considerable overlap in their functions” (Unfunded)	“Further, more than one class in qualitative research methods might be necessary to allow her to design and analyze qualitative research such as her focus groups” (Unfunded)
“Investigative team has demonstrated inter-professional collaboration, including strong record of funding, dissemination and publication of research findings" (Unfunded)	“Consultants [names redacted] provide expertise in participatory planning, and qualitative and mixed methods” (Unfunded)	“The PI has little experience in qualitative methods, and there is no track record of collaboration with the qualitative co-investigators to suggest an effective collaboration” (Unfunded)	“… it was noted that the application is not clear regarding who on the team has expertise in conducting focus groups” (Unfunded)
**Innovation**			
“Qualitative research can provide new insights regarding the impact of social and contextual influences on financial decisions which typically are ignored in quantitative research. Combining qualitative and quantitative methods is innovative” (Unfunded)	“will use photo voice and smartphones–these aspects contribute to the novel aspects of the application” (Funded)	“No novel methodologies are being proposed for this project. Mixed-methods approaches have been used in multiple other studies” (Unfunded)	“The study would not develop new methods. It would use conventional methods to compare one existing and widely-used method for obtaining data (EMA via smart phone) to another existing and widely used method (face-to-face interviews)” (Unfunded)
**Environment**			
None noted	“The primary mentor and two co-mentors are ideally suited to provide the mixed-methods training required for the candidate’s research plan” (Unfunded)	None noted	None noted

Concerning Investigators, a strength noted by reviewers was evidence of productivity as a mixed methods team. Evidence included previous collaborations with a record of successful funding and publications. While the principal investigator’s expertise in either qualitative or quantitative research was also noted as a strength, training or experience in mixed methods research was noted most frequently as a strength. Reviewers noted as a weakness overlapping roles without a clear and distinct delineation of co-investigator contributions. Reviewers looked for justification of any identified overlap. Aside from weaknesses noted about content area expertise, reviewers expressed concern whether needed expertise and skills were present in the team, which included skills or training in both qualitative and quantitative data collection and analysis methods.

Regarding Innovation, reviewers noted that the use of multiple data sources, including the use of mixed methods, was a strength to understand both mechanisms and contextual factors. Reviewers valued a clear description of why a mixed methods approach was most appropriate to accomplish the specific aims. In contrast, stating that the use of mixed methods was “innovative” was not compelling unless the investigator argued explicitly how methods were being combined in a novel way to accomplish the specific aims. We found no differences in comments about innovation between funded and unfunded grants.

#### K-series grants

We analyzed reviewer comments of K-series grants separately for each of the criteria appropriate to K awards (Table 4) because of their different focus on career development and mentored research. Regarding Significance, the mixed methods approach was viewed as a strength because the use of mixed methods was thought to yield richer information and allowed for an in-depth investigation. No weaknesses were noted for this criterion.

Concerning Investigators, several comments were made about proposed mentoring teams. Proposing a team of mentors representing both qualitative and mixed methods expertise, particularly with evidence that the team has worked together, was an important consideration. At the same time, lack of a statistician on the mentoring team was viewed as a major weakness. As for the applicant, a record of prior qualitative and mixed methods training through fellowships and training programs was viewed as a strength. A common weakness noted was the inadequacy of the applicant’s qualitative methods training plan.

With regard to Innovation, themes were consistent with those identified in the R-series grants. Namely, the novel use of integrated methods with a clear link to accomplishing the aims was a strength, while simply having qualitative and quantitative methods in the same project did not garner enthusiasm as innovative. Finally, in terms of Environment, only one comment was made about the researcher’s home institution being suited for conducting mixed methods research.

### Inductive themes

#### Human subjects concerns

A commonality in comments that emerged outside of our NIH-based coding scheme was ethical issues specific to mixed methods research. One main concern was the subject burden of participating in both qualitative and quantitative data collection for the proposed project. Reviewers were concerned that interviews could be time-intensive and burdensome for participants who may not be capable of maintaining the prolonged dialogue required. Another concern pertained to participant confidentiality and anonymity. Reviewers noted the risk of participants being identified in the qualitative phase from the information they provide during interviews. They also noted a need for special attention to participant protection if video recordings will be used in the study, especially if the purpose is to assess the skills or competence of the participants. Lastly, the informed consent process for a mixed methods project may be complex and lengthy because sampling or data collection strategies might be more complex when employing mixed methods. Reviewers expressed concern that the complexity of consent could be burdensome, and could affect enrollment rates.

#### Generalizability concerns

A few reviewers identified the limited generalizability of qualitative research as a weakness of the mixed methods application. Generalizability spans review criteria, including the approach and significance. One reviewer stated “the problem with this from a significance standpoint is that it is difficult to generalize any potential findings from this work because of this focus on individuals.” Reviewers also commented on the divergent nature of qualitative and quantitative sampling techniques, and the implications for generalizability when both techniques were used in a single study. Our database included summary statements from proposals in which the same sample was interviewed with both quantitative and qualitative components. In such a design, reviewers expressed concern that the sample size was too large to feasibly complete a qualitative study, while not large enough to be properly powered for quantitative analysis.

#### Reviewer attitudes toward mixed methods research

Several reviewers noted the mixed methods approach as a strength in general terms (“The mixed methods design is a strength of the proposed methodology”). One reviewer commended the investigators for specifying their knowledge and skills to leverage the full potential of mixed methods research. They stated, “the ongoing presence of qualitative study during the entire course of the proposed research is a particularly appealing aspect of this application. Ethnography is often ‘front-loaded’ or ‘back-loaded’ into mixed methods designs, but seldom is it thoroughly incorporated as an ongoing corrective to errors of interpretation and estimation. This application does that.”

### Differences in funded and unfunded grants

We analyzed the coded comments by investigating whether any noteworthy patterns emerged in terms of how reviewers appraised funded versus unfunded grant applications. Among funded applications, reviewers noted more strengths about the research team’s ability to integrate qualitative and quantitative strands due to their methodological expertise (“The excellent integration of the quantitative and qualitative components of the proposed study speaks well of this team’s qualifications and experience”). In unfunded applications, comments were in more general terms about research methods skills with no comments about the team’s ability to bring the two strands together (“She has extensive research in health behavior, … and mixed method research”). For the Approach, reviewers identified more strengths about the use of appropriate mixed methods designs in funded studies (n = 23 mentions) compared to unfunded studies (n = 11). Reviewers also had more positive comments about the description of integration in funded studies (n = 14) than in unfunded applications (n = 1); however, in both funded and unfunded applications, reviewers commented about the lack of integration details. Reviewers noted more weaknesses about the qualitative methods in unfunded studies (n = 35) compared to funded studies (n = 8). Reviewers identified more weaknesses about sampling strategies in unfunded studies (n = 38) than funded studies (n = 12). No patterns emerged for the Significance, Innovation, and Environment criteria.

## Discussion

Our sample was consistent with published research in which NCI, NIMH, and NIDA were among the institutes that fund the most grant applications using mixed methods.[8, 9] In this analysis of summary statements from mixed methods proposals, reviewers were positive about the value of mixed methods. Reviewers saw innovation when mixed methods approaches were specified clearly in the context of current knowledge for the particular research questions and had potential to yield a new understanding of a health outcome, patient experience, context, or mechanism of an intervention. Reviewers were most favorable about applications that demonstrated coherence among the aims and approach, provided detailed descriptions of methods, described how integration of qualitative and quantitative components would be achieved, and employed theoretical models. Regarding investigators, a track record of productivity among team members was important as was having requisite qualitative, biostatistics, and mixed methods expertise. Reviewers were troubled when methodological details were lacking or vague, particularly in qualitative sampling, qualitative analysis, or integration procedures. Reviewers noted concerns about human subjects and generalizability/transferability within proposed mixed methods studies.

Before discussing the implications of our findings for investigators writing mixed methods applications to NIH, the limitations of the study deserve comment. First, our sample was not representative of mixed methods proposals to NIH, or even of mixed methods proposals submitted by MMRTP scholars and consultants. By requesting critiques of unfunded proposals, we avoided only reviewing projects that were funded (e.g., through the use of NIH RePORTER). Given the favorable mean scores of the applications, even among the unfunded applications, our sample may have been among the best of mixed methods applications submitted to the NIH. Two applications were resubmitted for a second time (5.0%); these applications were submitted in the 1990s and early 2000s prior to NIH’s policy change in 2009 to allow only one resubmission. Second, the Best Practices[11, 12] were intended as guidance for investigators writing proposals to a specific organization, the NIH in the United States, and should not be construed as holding up a standard for all mixed methods health research.[21] Third, we were unable to examine the actual grant applications accompanying the summary statements to see precisely what was driving the reviewers’ comments or how revisions to applications were related to comments.

Table 5 summarizes recommendations for writing mixed methods grant applications, derived from findings. Despite limitations, our analysis reaffirms the idea that underlying conceptual frameworks driving the research aims and the procedures used to accomplish the specific aims should be clear and coherent. The job of the investigator in writing a proposal is to convey to the reviewer “how to think about” the specific use of mixed methods for accomplishing the specific aims at hand, as well as to clearly describe how the methods will be carried out (the “how to” of mixed methods) in service of those aims.[21] Investigators planning to use mixed methods should remember that reviewers are looking for a rationale that conveys the underlying logic behind procedures.

**Table 5:** Recommendations when writing mixed methods proposals for the NIH based on analysis of reviewer comments.

**Significance**
• Include a rationale for using mixed methods, why it is the best approach • Discuss the added value (the yield) of the mixed methods approach
**Investigators**
• Describe prior collaboration (funding/publication record) of the research team if applicable • Describe the PI’s engagement with mixed methods research (e.g. prior training, future training plans) ◦ If K-grant application, ensure qualitative training plan is adequate • Describe investigator roles ◦ Justify any overlapping roles ◦ Ensure needed expertise and skills are present in the team (qualitative expert, mixed methods expert, statistician)
**Innovation**
• Discuss why the combination of methods used to accomplish the aims is innovative ◦ Explain if the project will develop new methods
**Approach**
• Describe the type of mixed methods study design proposed and rationale for the identified Aims. • Ensure coherence among aims, research designs, and methods • Describe theoretical models or conceptual frameworks that guide the project • Describe the rationale behind sampling procedures ◦ Discuss both quantitative and qualitative sampling ◦ Discuss transferability (generalizability) in qualitative research ◦ Describe the relationship between the quantitative and qualitative samples (i.e. purposive sub-sample or identical samples) ◦ Discuss mixed methods sampling • Discuss qualitative data collection plan ◦ Describe data sources ◦ Consider unconventional data sources ◦ Describe how consistency in interviewing procedures will be achieved (e.g. interviewer training plans) ◦ Describe main domains for interviews with example questions ◦ Describe data management plans • Discuss qualitative data analysis plan ◦ Describe how consistency in coding procedures will be achieved (e.g. coding meeting plans) Discuss possible outcomes informed by relevant literature or investigator’s prior work • Discuss mixed methods integration Provide details on integration procedures In sequential designs, explain linkage between quantitative and qualitative procedures (e.g. how qualitative findings would inform intervention/instrument development) Explain mixed methods language/jargon (i.e. “mixing” or “integration”) in terms of procedures/techniques ◦ Describe when in the project integration occurs (could occur at multiple phases) ◦ Describe how differences in inferences made from quantitative and qualitative components will be reconciled • Discuss project feasibility ◦ Ensure enough time/resources are allocated to the qualitative component
**Environment**
• If K-series application, ensure mentors have mixed methods expertise • Describe the environment support for mixed methods research

### Writing an effective approach section

Synthesizing findings from our analysis, we now present recommendations for writing a research approach. Our findings highlight the importance of ‘educating the reviewers’ on the underpinnings of qualitative and mixed methods research. Keeping the audience in mind is particularly important when addressing generalizability of the proposed study findings because implicit disciplinary bias may operate when reviewers read applications.[22] Generalization is typically understood as a statistical notion that involves drawing broad inferences from specific circumstances.[23] However, criteria and discussions of generalizability in qualitative research are available,[24, 25] and given the multiplicity of qualitative approaches, citations regarding the goals of qualitative research can reassure reviewers. A number of reviewers in our sample applied the statistical idea of generalizability to the proposed qualitative aims and outcomes, and dismissed such methods as of limited value. As Myers asserts, some research questions are better answered by probing into personal accounts to understand the complexity of experiences within specific contexts, especially those surrounding deeply sensitive topics such as cancer, addiction, racial disparities and mental health.[26] Investigators need to avoid having the qualitative component come across as an “afterthought” or as a less rigorous aspect of research.

The sampling approach linking the quantitative and qualitative strands, as well as the qualitative data collection and analysis plans, attracted considerable attention from reviewers. Investigators should discuss the rationale for their sampling strategy, which should be guided by their research questions. For example, a small qualitative sample size may be justified if the purpose of that portion of the study is to achieve depth or analysis and understanding rather than generalizable knowledge. Yet with the incorporation of a relevant theory or conceptual framework, smaller sample qualitative results can serve as evidence that corroborates or does not corroborate the theory or framework, thus suggesting the transferability of these results to other similar samples or situations. Laying out the assumptions and rationale for the qualitative sample size helps anticipate criticism from a reviewer who is not familiar with the smaller sample sizes frequently employed in qualitative research.[27]

Investigators should comment on the relationship between the qualitative sample and quantitative sample. For example, a common sampling approach in health services research is one that begins with a probability sample followed by a purposive sub-sample for in-depth exploration.[15, 28] Such a design (sometimes called an “explanatory sequential” design[1]) is often used to provide a better understanding of patient or provider experiences. Mixed methods sampling has emerged as an independent sampling category, described as a procedure that combines qualitative and quantitative sampling strategies in creative ways to address the research questions at hand.[15, 28, 29] Investigators should refer to the mixed methods literature and pilot results when possible to reassure reviewers of the feasibility of the sampling strategy.

Our analysis revealed that reviewers were not as familiar with studies that use an identical sample for both qualitative and quantitative components (i.e. same sample of people participating in both strands of the study[29]). For example, having the same people participate in both strands of the study can be useful when there is a clear need to have qualitative and quantitative data from all the participants to expand understanding, compare, or perhaps relate qualitative to quantitative results. Such designs might be most suitable in intervention development or pilot studies in which the goal is evaluating feasibility or getting feedback from participants, where sample sizes may be smaller than required for a fully powered study.[30]

Investigators may be tempted to limit the description of their qualitative data collection and analysis plans in the interest of saving application space.[31] However, our research shows that reviewers look for detailed discussions, especially strategies that will enhance rigor (see[32]). For example, investigators should indicate how they intend to ensure consistency in interviewing and coding procedures by describing interviewer training and coding meeting plans. Applicants should also be sure to enumerate the main domains for interviews and perhaps provide examples of interview guide questions in a text box. Investigators should acknowledge the large volume of data the qualitative component will generate, as well as its time-intensive and exhaustive nature with clear data management plans (e.g., in the timeline and budget justification).

As recognized by one reviewer in our sample, a challenge that investigators face concerns the inductive nature of qualitative research that makes the methods difficult to predefine entirely in a proposal (see[33]). Describing the “directionality” of the research, coding, and data analytic plan, as guided by expected (deductively defined) and discovered (inductively generated) codes, will aid in convincing reviewers that the investigative team well understands the major thrust of the planned research, while also open to uncovering new data, allowing the qualitative evidence to “speak for itself.” Unexpected findings could emerge that threaten to derail the course of the research project, especially if subsequent aims rely on findings from the initial qualitative aim.[34] At times, identifying novel results that do not necessarily corroborate hypothesized results suggest that “something went wrong.” Conversely, interrogating such anomalous results may instead uncover an unexpected and novel finding that emerges from this serendipitous discovery, which may contribute to a new approach toward resolving the research problem under analysis.[35]

Investigators can offer a brief discussion about possible outcomes or alternative explanations by drawing on the relevant literature or their experience. In some proposals, the qualitative component of a study was described as ‘validating’ or ‘triangulating’ results from a quantitative component (see[35]). If triangulation is a strategy for the study, investigators should explain how they will reconcile differences in inferences made from quantitative and qualitative components (doing so may involve collecting additional data to reconcile differences or re-examining constructs[33]).

Unfavorable comments about integration were related to a lack of details on procedures. For example, in an exploratory sequential design (qualitative phase followed by a quantitative phase), investigators should describe how qualitative themes systematically map onto quantitative scales and items in developing instruments.[1] Because only one reviewer noted integration throughout all phases of the research process as a strength, it is unclear in which phase of the research reviewers felt integration needed to be described the most. Nevertheless, this comment deserves attention to highlight the importance of integration in a mixed methods study.[36] Investigators can be guided by literature describing how integration may be achieved at multiple stages of research.[36, 37] Moreover, PCORI standards for mixed methods emphasize a clear description of how methods are integrated across one or more stages of the research project, justification and details of sampling, and integration in data analysis, interpretation, and conclusions.[38]

### The mixed methods team

Challenges related to methodological and epistemological differences commonly surface in mixed methods research teams.[39] Lessons learned from prior collaborations can help resolve these issues during early stages of the project, which is of considerable value when project milestones must be met under a designated timeline. Effective collaborations through frequent meetings and open discussions of methodological issues have been linked to better engagement in integration of findings and the ability of the team to fully leverage the benefits of mixed methods research.[40] Investigators need to keep in mind the potential overlap of roles when putting together an interdisciplinary mixed methods team. Each team member’s complementary and not duplicative role should be clearly defined in the proposal. Investigators should also demonstrate their commitment to mixed methods research by noting their prior training or future plans to obtain training. Reviewers noted the principal investigator’s mixed methods experience as a strength more frequently than qualitative or quantitative expertise alone. Research on mixed methods team dynamics suggests that principal investigators who understand and value the integration aspect of mixed methods research convey their ability to facilitate team-level and project-level integration.[40, 41] Researchers submitting K-series applications should choose mentors with mixed methods expertise. Few reviewer comments concerned the Environment for mixed methods research, representing a lost opportunity for applicants to emphasize the institutional support for mixed methods.

### Making the case for innovation

Contrary to the belief that the cutting-edge nature of mixed methods itself is appealing to funding agencies, proposing mixed methods alone was not considered innovative by reviewers. However, reviewers noted that using a mixed methods approach to understand mechanisms and contexts not previously understood was innovative. Other examples using mixed methods that may be considered innovative include understanding why an intervention did or did not work,[42, 43] eliciting diverse perspectives on a problem,[44] or using a latent class transition model as a sampling frame for semi-structured interviews.[45] Convincing the reviewers that mixed methods is the best approach for addressing research questions may help ease concerns about the burden placed on research subjects whose participation is requested in both quantitative and qualitative components of the study.

## Conclusion

Mixed methods approaches are well suited for addressing the aims of health services and implementation research. Nevertheless, applicants should be careful to provide the assumptions behind the methods proposed so that reviewers who were primarily trained in quantitative methods from disciplines like epidemiology and statistics will understand the link between the specific aims and the need for mixed methods. Reviewers look for details about sampling, data collection and data analysis plans, and data integration procedures. Applicants should anticipate and mitigate reviewer concerns about potential drawbacks of mixed methods related to human subjects, time and resource intensity, and generalizability of findings. Our study provides some empirical guidance for investigators seeking to take full advantage of mixed methods to address pressing clinical and public health challenges.
